# Intense unimanual contractions increase muscle excitation and local metabolic activity in the resting contralateral forearm

**DOI:** 10.1113/EP094221

**Published:** 2026-10-27

**Authors:** Lee J. Hinkle, Britton C. Scheuermann, Carl J. Ade, Thomas J. Barstow, Joshua C. Carr

**Affiliations:** ^1^ Kinesiology Program, School of Health Sciences, College of Health and Human Sciences Kansas State University Manhattan Kansas USA

**Keywords:** functional laterality, muscle fatigue, near‐infrared, oxygen consumption, spectroscopy, surface electromyography

## Abstract

Intense unilateral muscle contractions evoke measurable activity within the contralateral neuroaxis, which can be detected with surface electromyographic activity in the resting homologous muscle. Physiological mirror activity (PMA), the unintentional increase in contralateral muscle excitation, has been implicated in cross‐limb interactions and adaptations. Despite longstanding observations of PMA, it remains unknown whether this low‐level muscle excitation influences local muscle metabolism. We addressed this question using a vascular occlusion test in 10 healthy adults. Surface electromyography and near‐infrared spectroscopy‐derived measures of tissue oxygen saturation and muscle oxygen consumption ($m{\dot{V}}_{O_{2}}$) were obtained from the resting left forearm during vascular occlusion at rest and during fatiguing unimanual contractions. PMA in the contralateral resting arm was greater during fatiguing unimanual contractions than during rest (mean difference: 8.9%, 95% CI: 4.1 to 13.8). This increase was accompanied by a steeper rate of tissue oxygen desaturation (mean difference: −0.132% s^−^^1^, 95% CI: −0.227 to −0.037) and greater $m{\dot{V}}_{O_{2}}$ (mean difference: 0.188 mL O_2_ min^−^^1^ 100 g^−^^1^, 95% CI: 0.057 to 0.320). Greater PMA was associated with both a faster rate of oxygen desaturation (*r* = −0.85, 95% CI: −0.96 to −0.46) and greater $m{\dot{V}}_{O_{2}}$ (*r* = 0.78, 95% CI: 0.28 to 0.94). These findings provide the first evidence that PMA is accompanied by increased local metabolic demand, consistent with a coupling between unintentional muscle excitation and oxygen extraction in the resting limb.

## Abstract (highlights)

- **What is the central question of this study?**
  Physiological mirror activity (PMA) accompanies intense unimanual contractions, yet its metabolic relevance remains unknown. Does PMA resulting from fatiguing unimanual contractions increase metabolic activity in the resting contralateral forearm?
- **What is the main finding and its importance?**
  This is the first evidence that PMA is accompanied by increased local metabolic activity in the resting muscle. Fatiguing unimanual contractions increased contralateral muscle excitation, accelerated oxygen desaturation, and roughly doubled muscle oxygen consumption in the resting forearm. In exploratory analyses, greater PMA was associated with greater oxygen desaturation and consumption.

## 1 INTRODUCTION

Intense unilateral motor actions increase excitation in homologous muscles of the resting contralateral limb. This response, classically termed motor irradiation (Hellebrandt, 1951; Hopf et al., 1974) and more recently physiological mirror activity (Maudrich et al., 2018), reflects bilateral interactions within the motor system that become increasingly evident as voluntary drive rises (Carson, 2005; Welniarz et al., 2015). Although unilateral actions are behaviorally lateralized, their neural control engages bilateral motor networks in which common supraspinal drive and interhemispheric interactions shape the excitability of both motor pathways (Cabibel et al., 2020; Carson, 2005, 2020; Chettouf et al., 2020). The strongest evidence places these interactions primarily at the cortical level, where excitatory drive from motor regions upstream of the primary motor cortex and transcallosal inhibitory and facilitatory processes regulate unintended activation of the opposite hemisphere and its descending output (Cabibel et al., 2020; Carson, 2005, 2020; Chettouf et al., 2020).

The resulting physiological mirror activity can be quantified using surface electromyography (sEMG) (Yensen, 1965) by expressing the unintentional muscle excitation recorded from the resting homologous muscle during unilateral contraction relative to that same muscle's maximal muscle excitation, obtained during a maximal contraction of the same limb. This normalized value, termed associated sEMG activity, represents the fraction of maximal muscle excitation expressed involuntarily during a unilateral task. The magnitude of the associated sEMG activity emanating from the resting contralateral homologous muscle scales with the relative intensity of unilateral motor output and also rises with fatiguing contractions (Maudrich et al., 2019; Sehm et al., 2010). With associated sEMG activity in the resting limb reaching ∼10–40% of maximal muscle excitation, this degree of involuntary excitation would be expected, in principle, to increase motor unit activity, twitch force generation, and as a result, some degree of metabolic demand. Such changes would be expected to manifest as increased oxygen consumption and reduced tissue oxygen saturation within the active musculature. However, whether this low‐level involuntary excitation is sufficient to produce measurable changes in local muscle metabolism remains unknown. To this end, establishing whether a coupling between physiological mirror activity and local metabolic activity exists would provide evidence of an energetic consequence of incomplete lateralization of motor output and lay the groundwork for future investigations into understudied mechanisms underlying cross‐limb adaptations (Hortobagyi et al., 2011; Reissig et al., 2015).

Therefore, the purpose of the present study was to determine whether physiological mirror activity during maximal unimanual contractions increases local muscle deoxygenation and oxygen consumption in the resting contralateral forearm. We addressed this question using a vascular occlusion test, in which cuff‐induced arrest of arterial inflow allows local oxygen consumption to be estimated from the rate of near‐infrared spectroscopy (NIRS)‐derived tissue deoxygenation (Barstow, 2019; Hammer et al., 2019). We recorded sEMG and NIRS‐derived tissue oxygen saturation ($S_{tO_{2}}$) and muscle oxygen consumption ($m{\dot{V}}_{O_{2}}$) from the resting left forearm during occlusion under two conditions: at rest and during repeated maximal handgrip contractions. Our hypothesis was that fatiguing unimanual contractions would increase associated sEMG activity (%AA), accelerate the decline in tissue saturation, and increase muscle oxygen consumption in the resting contralateral forearm compared with control conditions. Additionally, we explored whether the magnitude of associated sEMG activity was related to the reduction in tissue oxygen saturation and the increase in muscle oxygen consumption to determine whether there is a physiological coupling between involuntary contralateral muscle excitation and local muscle metabolism.

## 2 METHODS

This study received ethical approval from Kansas State University's Institutional Review Board for Human Subjects Research (IRB‐12918), and all subjects signed an Informed Consent document. The study adhered to the ethical principles of the *Declaration of Helsinki*, except for prospective registration in a publicly accessible database.

### 2.1 Experimental design

This study employed a within‐subject repeated‐measures design to determine whether physiological mirror activity increases metabolic activity in the resting contralateral forearm during fatiguing unimanual contractions. Participants completed two experimental conditions involving a 3‐min vascular occlusion test of the left (non‐dominant) forearm with simultaneous NIRS and sEMG recordings. During the control condition, the vascular occlusion test was performed at rest. During fatiguing unimanual contractions the vascular occlusion test was performed while participants concurrently completed repeated maximal unimanual handgrip contractions with the right hand throughout the occlusion period. The order of conditions was fixed to minimize the influence of fatigue‐induced carryover effects on the control condition (e.g., cardiovascular and autonomic perturbations). All testing was completed during a single laboratory visit, with a 10‐min washout period between conditions, consistent with previous work (Costalat et al., 2026; McLay et al., 2016). The primary outcomes were tissue oxygen saturation slope (% s^−^^1^; a negative value indicating desaturation) during the vascular occlusion test and the derived estimate of muscle oxygen consumption, both used as indices of local metabolic activity, as well as associated sEMG activity, all recorded from the resting left forearm. A graphical overview of the experimental design is shown in Figure 1.

**FIGURE 1:**
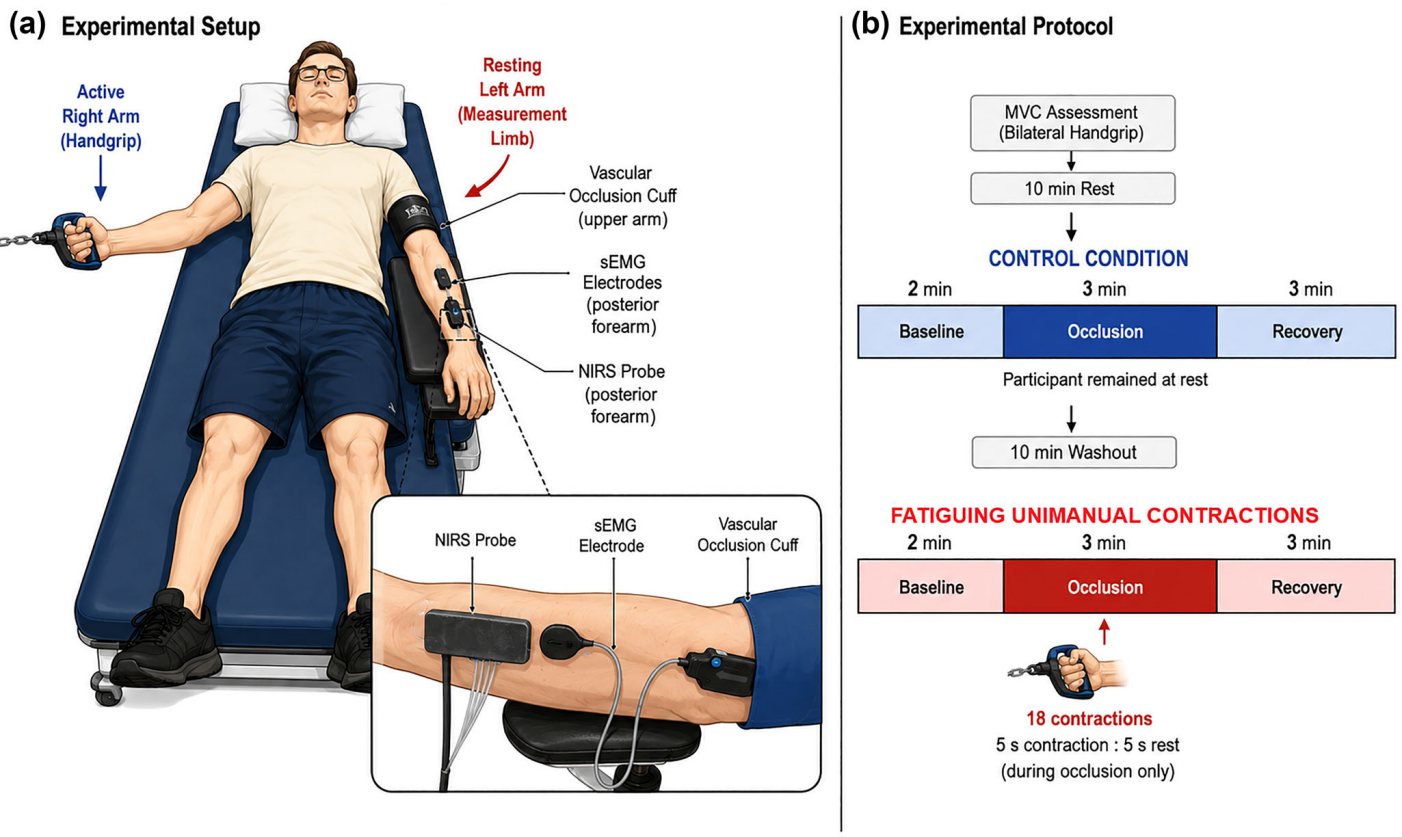
Experimental setup and study protocol. Experimental design and instrumentation used to assess physiological mirror activity and local metabolic responses in the resting contralateral forearm during fatiguing unimanual contractions. (a) Experimental setup. Participants were positioned supine on a padded treatment table. The dominant (right) hand performed intermittent maximal handgrip contractions during the fatigue condition, whereas the non‐dominant (left) forearm remained at rest throughout both experimental conditions. Surface EMG sensor and the NIRS probe were positioned over the left extensor carpi radialis, and a pneumatic cuff placed around the proximal left upper arm was inflated to induce vascular occlusion. The inset illustrates the relative placement of the sEMG sensor, NIRS probe, and vascular occlusion cuff on the posterior aspect of the left forearm. (b) Experimental protocol. Following maximal voluntary contraction (MVC) assessment and a 10‐min rest period, participants completed a control vascular occlusion test consisting of a 2‐min baseline, 3‐min vascular occlusion, and 3‐min recovery while remaining at rest. After a 10‐min washout period, participants completed an identical vascular occlusion protocol while simultaneously performing 18 intermittent maximal handgrip contractions (5‐s contraction: 5‐s rest duty cycle) with the right hand during the 3‐min occlusion period.

### 2.2 Participants

Ten healthy adults completed the study (*n* = 10; 4 females, 2 of whom used hormonal contraceptives). For females, menstrual cycle phase was not controlled because testing was completed during a single laboratory visit. Participant characteristics were age 22.2 ± 5.5 years and body mass index 25.2 ± 2.9 kg m^−^^2^. Eligibility was determined using a medical history questionnaire.

### 2.3 Experimental procedures

Experimental testing began with the assessment of maximal handgrip force. All procedures were performed with participants positioned supine on a padded treatment table. Maximal handgrip force and maximal sEMG amplitude were obtained from the left forearm, and maximal sEMG amplitude was used to normalize the associated contralateral sEMG activity, as described below. After these initial measurements, participants rested for 10 min before completing the control vascular occlusion test. Each vascular occlusion test consisted of a 2‐min baseline period, a 3‐min occlusion period, and a 3‐min recovery period. Following a 10‐min washout period, participants completed the fatiguing unimanual contractions condition using the same vascular occlusion test parameters. sEMG and NIRS from the left resting forearm were sampled continuously during the 3‐min occlusion period in both conditions. The analyses focused on the wrist extensors because associated sEMG activity has been shown to be greater in the extensors than in the flexors during unimanual handgrip (Carr et al., 2021).

### 2.4 Control and fatiguing unimanual contractions conditions

During the control condition, participants remained at rest on the treatment table while viewing a visual force feedback display set at 0% and were instructed to remain still throughout the vascular occlusion test. During the fatiguing unimanual contractions condition, participants performed intermittent maximal handgrip contractions with the right hand using a 50% duty cycle (5 s contraction, 5 s rest) throughout the vascular occlusion test, for a total of 18 contractions. Participants received visual feedback of both their maximal force reference and active force output and were instructed to contract as close to their maximal force as possible. Strong verbal encouragement was provided throughout the task. In both conditions, participants received no instruction regarding the left forearm other than to remain relaxed and focus their attention on the force tracing.

### 2.5 Maximal voluntary contraction and force measurement

Maximal voluntary contraction (MVC) force was obtained during unimanual handgrip using a modified handgrip dynamometer attached to a tension‐compression load cell (Interface Inc., Scottsdale, AZ, USA; model SSM‐AJ‐500), similar to previous work (Carr et al., 2021). The dominant hand was assessed first, followed by the non‐dominant hand. Following a brief familiarization and warm‐up, maximal handgrip force was obtained during a 5 s MVC trial with real‐time visual force feedback displayed on a monitor during all testing contractions. Right‐hand MVC was extracted for each of the 18 contractions to verify performance fatigability.

### 2.6 Surface electromyography

Before sensor placement, B‐mode ultrasonography was used to identify the extensor carpi radialis (ECR) muscle belly of the left forearm to guide sensor placement. For most participants, the sensor was approximately 30% of the distance between the medial epicondyle of humerus and the styloid process of ulna. The skin was then prepared with isopropyl alcohol. Surface electromyography (sEMG) was recorded from the ECR using a four‐channel Galileo sensor (Delsys Inc., Natick, MA) with 2.5 mm diameter EMG contact area, positioned near the centroid of the muscle. sEMG signals were sampled at 2222 Hz and band‐pass filtered at 20–450 Hz. sEMG amplitude was quantified as the root mean square (RMS) using a 250‐ms moving window. RMS values were averaged across the four channels within the sensor to obtain a representative signal for the ECR. Maximal sEMG amplitude was defined as the highest 500‐ms mean RMS value obtained during maximal voluntary handgrip testing, and associated sEMG activity during the experimental conditions was expressed relative to the sEMG activity during ipsilateral maximal voluntary contraction. Associated sEMG activity was calculated for each of the 18 contractions during the fatiguing unimanual contractions condition and a time‐locked comparison of the same time intervals were captured from the control condition using custom R scripts to obtain eighteen 5‐s bins for both conditions. Each 5‐s bin contained the average sEMG activity for that time epoch and were then averaged across bins to obtain a single representative measure for each condition. More specifically, the associated sEMG activity was quantified as the ratio of sEMG amplitude recorded from the left extensor carpi radialis during the control and right hand fatiguing unimanual contractions conditions (EMG_resting_) to sEMG amplitude recorded from the same left forearm during left‐hand maximal contractions (EMG_ipsilateralMVC_). Associated sEMG activity (%AA) was calculated and expressed as a percentage:

$$\mathrm{Associated}\;\mathrm{EMG}\;\mathrm{activity}\;\left(\%\mathrm{AA}\right)=\frac{\mathrm{EM}G_{\mathrm{resting}}}{\mathrm{EM}G_{\mathrm{ipsilateralMVC}}}\;\times 100$$

### 2.7 Near‐infrared spectroscopy, vascular occlusion and outcome derivation

Tissue oxygen saturation ($S_{tO_{2}}$) was measured using a wireless continuous‐wave NIRS device (OxiplexTS; ISS Inc., Champaign, IL, USA). The probe was positioned over the left forearm extensor compartment, aligned with the extensor carpi radialis muscle belly at approximately 30% of the distance from the lateral epicondyle of the humerus to the styloid process of the radius, and placed distal to the sEMG sensor. $S_{tO_{2}}$ signals were sampled at 2 Hz. Vascular occlusion was induced via inflation of a rapidly inflatable pneumatic cuff (E20/AG101, D. E. Hokanson, Inc., Bellevue, WA, USA) and placed proximally on the upper arm to 300 mmHg. Successful occlusion was confirmed by a rapid decline in the $S_{tO_{2}}$ signal following cuff inflation. During both conditions, $S_{tO_{2}}$ signals were low‐pass filtered using a 10th‐order Butterworth filter with a cutoff frequency of 0.1 Hz. Signals were visually inspected for quality, and all recordings were retained for analysis. $S_{tO_{2}}$ slope (% s^−^^1^), was calculated as the linear regression slope of $S_{tO_{2}}$ during the first 60 s of vascular occlusion using least‐squares estimation (Barstow, 2019; Gomez et al., 2008). More negative slopes indicate a faster decline in $S_{tO_{2}}$ and are interpreted as greater local oxygen extraction under ischaemic conditions.

Muscle oxygen consumption ($m{\dot{V}}_{O_{2}}$; mL O_2_ min^−^^1^ 100 g tissue^−^^1^) was estimated from the initial rate of change in the [haem] difference signal ([haem]diff = oxy‐[haem] − deoxy‐[haem]) during the first 60 s of occlusion, quantified as the linear regression slope of [haem]diff versus time (Hammer et al., 2019). Because the OxiplexTS system reports chromophore concentrations in haemoglobin‐equivalent units, whereas myoglobin contributes substantially to NIRS signals in skeletal muscle, the output variables were multiplied by a factor of 4 to restore total [haem] units before calculation of [haem]diff and $m{\dot{V}}_{O_{2}}$ (Craig et al., 2017; Davis & Barstow, 2013; Hammer et al., 2018). Accordingly, the slope of [haem]diff was converted to $m{\dot{V}}_{O_{2}}$ using established assumptions regarding haemoglobin oxygen‐binding capacity, tissue density and molar gas volume (Hammer et al., 2019).

### 2.8 Statistical analysis

An a priori power analysis was not performed because directly comparable data for the present experimental model and outcome measures are not available. The sample size was determined based on preliminary pilot data within our laboratory that displayed large effects for the steeper $S_{tO_{2}}$ slope during fatiguing unimanual contractions. Within‐subject differences between conditions were examined using Student's two‐tailed paired *t*‐test between conditions for all outcomes, with results reported as mean differences and corresponding 95% confidence intervals. The primary analyses evaluated differences in tissue oxygen saturation slope, muscle oxygen consumption and associated sEMG activity between conditions. Exploratory analyses evaluated the association between associated sEMG activity and tissue oxygen saturation slope as well as muscle oxygen consumption using Pearson's product‐moment correlation coefficient; these bivariate analyses were selected to characterize the relationship between the magnitude of physiological mirror activity and each metabolic response independently. Effect sizes are reported as Hedges’ *g*. Statistical significance was set at α = 0.05. All analyses were performed in R version 4.5.1 (R Core Team, 2023).

## 3 RESULTS

Ten participants completed the study (*n* = 10; 4 female). All participants completed both experimental conditions and were included in the analyses.

### 3.1 Primary outcomes

Associated EMG activity averaged 13.4 ± 7.0%AA during the fatiguing unimanual contractions and 4.5 ± 2.8%AA during the control condition. The mean paired difference was 8.91%AA (95% CI: 4.06 to 13.76; *t*(9) = 4.15, *P* = 0.002, *g* = 1.20), with all but one participant exhibiting greater associated activity during the fatiguing unimanual contractions (Figure 2a). In parallel with these neuromuscular changes, the tissue oxygen saturation slope during the first 60 s of arterial occlusion averaged −0.258 ± 0.130% s^−^^1^ during the fatiguing unimanual contractions condition and −0.126 ± 0.067% s^−^^1^ during the control condition. The mean paired difference was −0.132% s^−^^1^ (95% CI: −0.227 to −0.037; *t*(9) = −3.14, *P* = 0.012, *g* = −0.91), consistent with greater oxygen extraction in the resting limb during fatiguing unimanual contractions (Figure 2b). To determine whether the greater rate of oxygen desaturation was accompanied by increased metabolic demand, muscle oxygen consumption ($m{\dot{V}}_{O_{2}}$) was examined. Muscle oxygen consumption in the resting contralateral extensor carpi radialis averaged 0.393 ± 0.202 mL O_2_ min^−^^1^ 100 g^−^^1^ during fatiguing unimanual contractions and 0.204 ± 0.128 mL O_2_ min^−^^1^ 100 g^−^^1^ during the control condition. The mean paired difference was 0.188 mL O2 min^−1^ 100 g^−1^ (95% CI: 0.057 to 0.320; *t*(9) = 3.24, *P* = 0.010, *g* = 0.94), with all but one participant exhibiting greater muscle oxygen consumption during fatiguing unimanual contractions (Figure 2c). Right‐hand MVC declined from the first to the eighteenth contraction (mean decline: −24.3 ± 11.7%; 95% CI: −32.6% to −15.9%), demonstrating performance fatigability across the protocol (Enoka & Duchateau, 2016).

**FIGURE 2:**
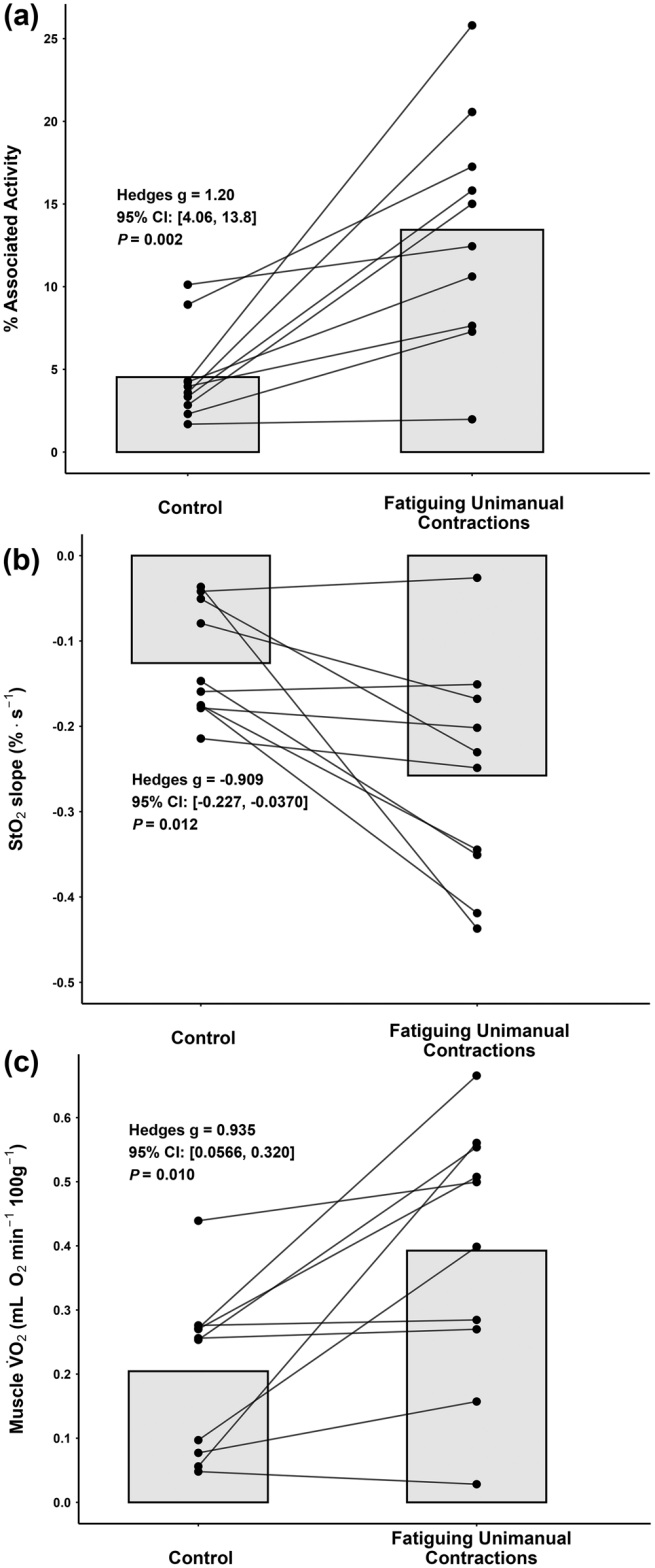
(a) Associated sEMG activity (%AA) in the left extensor carpi radialis during control and fatiguing unimanual contractions. Individual participant responses (*n* = 10) are shown as paired data points connected by lines, with group means displayed as bars. Associated sEMG activity was greater during fatiguing unimanual contractions (13.4 ± 7.0%AA) than during the control condition (4.5 ± 2.8%AA), with a mean paired difference of 8.91%AA (95% CI: 4.06 to 13.76; *t*(9) = 4.15, *P* = 0.002; *g* = 1.20). (b) Tissue oxygen saturation ($S_{tO_{2}}$) slope (% s^−^^1^) in the left (contralateral) extensor carpi radialis during control and fatiguing unimanual contractions. Individual participant responses (*n* = 10) are shown as paired data points connected by lines, with group means displayed as bars. The $S_{tO_{2}}$ slope was more negative during fatiguing unimanual contractions (−0.258 ± 0.130% s^−^^1^) than during the control condition (−0.126 ± 0.067% s^−^^1^), with a mean paired difference of −0.132% s^−^^1^ (95% CI: −0.227 to −0.037; *t*(9) = −3.14, *P* = 0.012; *g* = −0.91). (c) Muscle oxygen consumption ($m{\dot{V}}_{O_{2}}$; mL O_2_ min^−^^1^ 100 g^−^^1^) in the left (contralateral) extensor carpi radialis during control and fatiguing unimanual contractions. Individual participant responses (*n* = 10) are shown as paired data points connected by lines, with group means displayed as bars. Muscle oxygen consumption was greater during fatiguing unimanual contractions (0.393 ± 0.202 mL O_2_ min^−^^1^ 100 g^−^^1^) than during the control condition (0.204 ± 0.128 mL O_2_ min^−^^1^ 100 g^−^^1^), with a mean paired difference of 0.188 mL O_2_ min^−^^1^ 100 g^−^^1^ (95% CI: 0.057 to 0.320; *t*(9) = 3.24, *P* = 0.010; *g* = 0.94).

### 3.2 Relationship between associated activity and metabolic responses

Because associated sEMG activity, $S_{tO_{2}}$ slope and muscle oxygen consumption differed between conditions, additional analyses were performed to determine whether the magnitude of contralateral muscle excitation was related to local metabolic responses during the fatiguing unimanual contractions. Associated sEMG activity was strongly correlated with $S_{tO_{2}}$ slope, such that greater contralateral excitation was accompanied by a more rapid decline in tissue oxygen saturation (*r* = −0.845, 95% CI: −0.962 to −0.459, *P* = 0.002). Associated sEMG activity was also positively correlated with muscle oxygen consumption (*r* = 0.775, 95% CI: 0.284 to 0.944, *P* = 0.008), indicating that participants exhibiting greater physiological mirror activity also demonstrated greater oxygen consumption in the resting contralateral forearm (Figure 3a,b). To account for between‐condition differences, change scores (fatiguing unimanual contractions minus control) were also examined and supported these associations: Δ%AA correlated with Δ$S_{tO_{2}}$ slope (*r* = −0.73, *P* = 0.016) and Δ$m{\dot{V}}_{O_{2}}$ (*r* = 0.73, *P* = 0.017).

**FIGURE 3:**
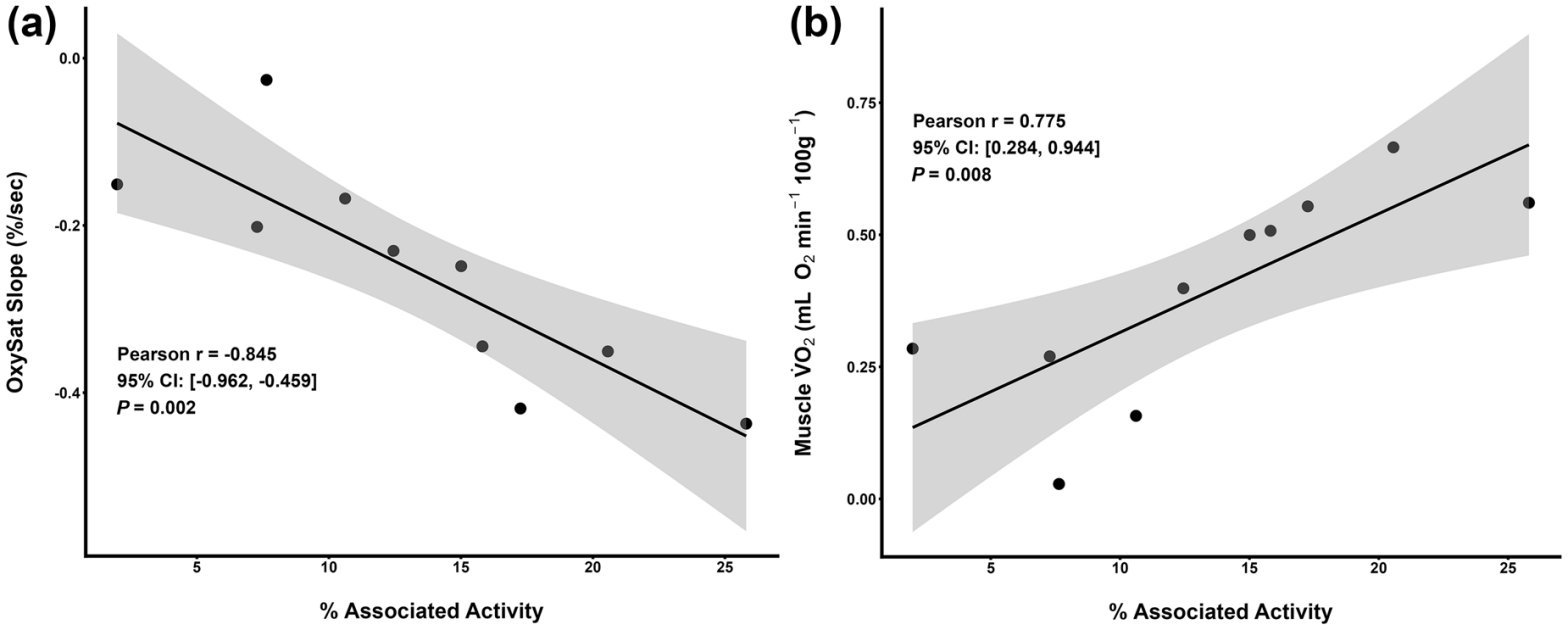
Associations between physiological mirror activity and local metabolic responses during fatiguing unimanual contractions. Each point is one participant's value during the fatiguing unimanual contractions condition (*n* = 10 participants). (a) Relationship between associated sEMG activity (%AA) and $S_{tO_{2}}$ slope in the resting contralateral extensor carpi radialis. Greater associated activity was associated with a more negative $S_{tO_{2}}$ slope (*r* = −0.845, 95% CI: −0.962 to −0.459; *P* = 0.002). (b) Relationship between associated sEMG activity (%AA) and $m{\dot{V}}_{O_{2}}$. Greater associated activity was associated with higher muscle oxygen consumption (*r* = 0.775, 95% CI: 0.284 to 0.944; *P* = 0.008). Shaded regions represent 95% confidence intervals around the fitted regression lines.

## 4 DISCUSSION

The present study examined whether physiological mirror activity during maximal unimanual contractions is accompanied by measurable local muscle deoxygenation in the resting contralateral forearm. Using vascular occlusion while recording sEMG and NIRS‐derived tissue oxygen saturation and muscle oxygen consumption, fatiguing unimanual contractions increased associated EMG activity, accelerated the decline in tissue oxygen saturation and increased muscle oxygen consumption in the resting contralateral forearm compared with the control condition. This is the first evidence that EMG‐confirmed physiological mirror activity is accompanied by increased local oxygen consumption in the resting muscle. Although some increase in metabolic demand with muscle excitation is intuitive, the magnitude of this response is noteworthy. A seemingly trivial increase in involuntary muscle excitation (∼9%) was accompanied by an approximately twofold increase in muscle oxygen consumption (0.20 to 0.39 mL O_2_ min^−^^1^ 100 g^−^^1^). Therefore, even relatively low amplitude physiological mirror activity carries a measurable local metabolic consequence. Whether the energetic cost is sufficient to influence muscle function or adaptation remains to be determined and is briefly speculated upon below. Collectively, these findings provide a physiological basis for considering mirror activity as more than an electrophysiological by‐product of incomplete motor lateralization.

In the active limb, increasing muscle activation is accompanied by increased oxygen utilization and extraction rate (Alfonsi et al., 1999; Daniel et al., 2023), with metabolic demand scaling as a function of contraction intensity (Praagman et al., 2003). Therefore, it is reasonable to expect that measurable involuntary excitation in the contralateral resting musculature would likewise impose a local energetic demand, even if the magnitude of activation remains substantially below that of the active limb. Previous studies demonstrated contralateral vascular and oxygenation responses during unimanual exercise (Asahara et al., 2020; Eklund et al., 1974; Ishii et al., 2017), suggesting that unilateral exercise can influence the resting limb. However, because systemic circulation remained intact in these studies, changes in contralateral oxygenation could reflect centrally mediated vascular responses, altered perfusion or broader cardiovascular adjustments rather than local metabolic activity within the resting musculature itself. Consequently, these studies demonstrated that unilateral exercise influences the resting limb but could not isolate whether physiological mirror activity was accompanied by increased local oxygen utilization. The present study sought to determine whether physiological mirror activity is accompanied by local oxygen utilization within the same contralateral tissue exhibiting involuntary excitation. By applying vascular occlusion to the resting forearm, oxygen delivery and venous washout were constrained, allowing changes in tissue oxygenation and muscle oxygen consumption to primarily reflect local oxygen extraction within the ischaemic tissue volume (Barstow, 2019; Gomez et al., 2008; Lucero et al., 2018). Under these conditions, the accelerated decline in contralateral $S_{tO_{2}}$ and the increase in $m{\dot{V}}_{O_{2}}$ observed during fatiguing unimanual contractions are consistent with greater local oxygen extraction and oxygen consumption within the resting forearm. Notably, we observed this with modest associated activity (13.4%AA) in a muscle in which no contraction was intended, resulting in a near doubling of $m{\dot{V}}_{O_{2}}$.

We observed greater associated sEMG activity alongside steeper declines in contralateral tissue oxygen saturation and greater muscle oxygen consumption, suggesting that larger magnitudes of involuntary muscle excitation are accompanied by greater local oxygen extraction and oxygen consumption within the resting forearm. Although the present design cannot determine the precise metabolic processes responsible for this response, the most parsimonious explanation is that increased associated EMG activity reflects greater motor unit activity in the resting musculature. Even at low amplitudes, repeated motor unit discharge and low‐level twitch activity would be expected to increase metabolic activity due to the contractile activity within recruited fibres. There are three lines of evidence in support of this. First, the vascular occlusion test supports this interpretation because, in the absence of blood flow, greater local oxygen utilization would be reflected as accelerated $S_{tO_{2}}$ desaturation. Second, $m{\dot{V}}_{O_{2}}$ was greater during fatiguing unimanual contractions, providing an independent estimate of increased local oxygen consumption within the resting forearm. Third, the associations between %AA and both $S_{tO_{2}}$ slope and $m{\dot{V}}_{O_{2}}$ further suggests that the magnitude of involuntary muscle excitation was linked to the local metabolic response. Importantly, given the predominantly cortical and supraspinal basis of physiological mirror activity (Carson, 2005), these findings demonstrate that intense unilateral volitional drive is accompanied by measurable metabolic demand in the resting contralateral musculature. Thus, physiological mirror activity is not solely an electrophysiological manifestation of bilateral motor‐system interactions, the neural drive that escapes complete lateralization has a quantifiable peripheral consequence. This extends physiological mirror activity beyond neural ‘spillover’ alone and establishes a coupling between unintended contralateral excitation and local muscle metabolism.

Physiological mirror activity has long been considered a candidate mechanism contributing to the cross‐education response, and prior work has attempted to determine whether this unintended excitation accounts for contralateral adaptations (Hortobagyi et al., 2011; Hortobágyi et al., 1997). However, the evidence remains mixed as some show that mirror activity predicts cross‐limb adaptations in some short‐term paradigms (Reissig et al., 2015), whereas others suggest that associated EMG activity is insufficient to account for contralateral strength gains (Hortobagyi et al., 2011) or the preservation of strength and muscle size during immobilization (Andrushko, Gould et al., 2018; Andrushko, Lanovaz et al., 2018). This dissociation suggests that mirror activity is unlikely to operate as a direct training stimulus, but it may still represent one component of a broader cross‐limb response. The present findings extend this notion by showing that intense, repeated unilateral contractions evoke a local metabolic response within the contralateral resting musculature. Accordingly, repeated unilateral contractions may impose small but recurring neural‐metabolic demands in the contralateral limb. Over time, this energetic stress and repeated metabolic activation could plausibly interact with centrally mediated motor adaptations and local cross‐limb molecular signalling to contribute to adaptive processes within the untrained musculature (Hendy & Lamon, 2017). This possibility is consistent with preclinical evidence that unilateral treatments can evoke contralateral skeletal muscle responses, including immune‐cell signalling (Waters‐Banker et al., 2014) along with enhanced tissue remodelling and protein turnover in the opposite limb following mechanotherapy (Miller et al., 2018), and immune‐ and stress‐response transcriptional pathways following unilateral electrical stimulation (Shirai et al., 2025). This speculation provides a physiological framework by which chronic unilateral training could influence the untrained limb through the coupling of central motor drive, local contralateral oxygen utilization and cross‐limb molecular signalling. Defining these mechanisms in unilateral training paradigms will further advance our understanding of adaptive cross‐limb responses.

### 4.1 Methodological considerations

We note several considerations when interpreting the present findings. First, NIRS does not directly measure metabolism, but rather detects changes in the absorption characteristics of haem‐containing chromophores within the sampled tissue volume. Accordingly, both tissue oxygen saturation ($S_{tO_{2}}$) and the derived estimate of muscle oxygen consumption ($m{\dot{V}}_{O_{2}}$) should be interpreted as indirect indices of local oxygen utilization rather than direct measurements of metabolic flux. Further, NIRS‐derived signals represent mixed tissue contributions that may include skin, adipose tissue, microvasculature, and skeletal muscle. Therefore, we interpret the $S_{tO_{2}}$ slope primarily as an indirect estimate of local oxygen extraction and $m{\dot{V}}_{O_{2}}$ as an estimate of local aerobic metabolic rate derived from NIRS‐based changes in haem oxygenation during vascular occlusion (Hammer et al., 2019; Lucero et al., 2018). The use of vascular occlusion was an intentional methodological approach designed to constrain oxygen delivery and minimize systemic blood flow influences, thereby improving interpretation of local oxygen extraction within the resting forearm. However, even under occlusion, both $S_{tO_{2}}$ slope and $m{\dot{V}}_{O_{2}}$ remain NIRS‐derived estimates of local oxygen utilization rather than direct measures of tissue metabolism. The fixed‐order experimental design minimized fatigue carryover effects between conditions (Costalat et al., 2026; Gomez et al., 2008) but does not fully exclude potential order effects. In addition, the modest sample size should be considered when interpreting the breadth of these findings, including whether sex influences the responses (Doix et al., 2018; Sars et al., 2018), although the within‐subject consistency, large effect sizes and relatively narrow confidence intervals support the robustness of the observed responses. Despite these considerations, the combined use of vascular occlusion, sEMG and NIRS provides convergent evidence supporting an association between physiological mirror activity and increased local oxygen extraction and oxygen consumption in the resting forearm.

### 4.2 Conclusion

We show that physiological mirror activity during fatiguing unimanual contractions is accompanied by accelerated deoxygenation and increased muscle oxygen consumption in the resting contralateral forearm, consistent with enhanced local metabolic activity. We provide the first evidence linking EMG‐confirmed physiological mirror activity to a local metabolic response and quantify the magnitude of this energetic consequence, with $m{\dot{V}}_{O_{2}}$ nearly doubling during the fatiguing unimanual condition. In exploratory analyses, the increase of unintentional muscle excitation (%AA) was associated with a steeper decline in $S_{tO_{2}}$ and greater $m{\dot{V}}_{O_{2}}$. These strong associations suggest that physiological mirror activity – an established feature of intense unilateral muscle contraction – produces measurable metabolic consequences in the contralateral homologous pair experiencing involuntary excitation. These findings extend our understanding of cross‐limb interactions by demonstrating coupled neural and metabolic responses in the resting limb during fatiguing unimanual contractions, providing new insight into bimanual coupling and potential adaptive mechanisms involved in the cross‐education response.

## AUTHOR CONTRIBUTIONS

Conceptualization: Britton C. Scheuermann, Carl J. Ade, Thomas J. Barstow, Joshua C. Carr; Methodology: Lee J. Hinkle, Britton C. Scheuermann, Carl J. Ade, Thomas J. Barstow, Joshua C. Carr; Formal analysis and investigation: Lee J. Hinkle, Britton C. Scheuermann, Joshua C. Carr; Writing—original draft preparation: Lee J. Hinkle, Joshua C. Carr; Writing—review and editing: Lee J. Hinkle, Britton C. Scheuermann, Carl J. Ade, Thomas J. Barstow, Joshua C. Carr; Resources: Britton C. Scheuermann, Carl J. Ade, Thomas J. Barstow, Joshua C. Carr; Supervision: Joshua C. Carr. All authors reviewed and edited the final version for submission. All authors have read and approved the final version of this manuscript and agree to be accountable for all aspects of the work in ensuring that questions related to the accuracy or integrity of any part of the work are appropriately investigated and resolved. All persons designated as authors qualify for authorship, and all those who qualify for authorship are listed.

## CONFLICT OF INTEREST

The authors declare no conflicts of interest.

## FUNDING INFORMATION

No funding was received supporting this work.

## GENERATIVE AI STATEMENT

Generative AI (Chat GPT 5.0‐5.5) was used to ensure communication clarity and speed coding of statistical solutions.

## Data Availability

Data are stored on a public repository with access through the corresponding author.
